# Multiple factors influence compliance with colorectal cancer staging recommendations: an exploratory study

**DOI:** 10.1186/1472-6963-8-34

**Published:** 2008-02-06

**Authors:** Anna R Gagliardi, Frances C Wright, Mahmoud A Khalifa, Andrew J Smith

**Affiliations:** 1 Department of General Surgery, Sunnybrook Health Sciences Centre, Toronto, Canada; 2 Department of Pathology, Sunnybrook Health Sciences Centre, Toronto, Canada

## Abstract

**Background:**

For patients with colorectal cancer (CRC) retrieval by surgeons, and assessment by pathologists of at least 12 lymph nodes (LNs) predicts the need for adjuvant treatment and improved survival. Different interventions (educational presentation, engaging clinical opinion leaders, performance data sent to hospital executives) to improve compliance with this practice had variable results. This exploratory study examined factors hypothesized to have influenced the outcome of those interventions.

**Methods:**

Semi-structured interviews were conducted with 26 surgeons and pathologists at eleven hospitals. Clinicians were identified by intervention organizers, public licensing body database, and referral from interviewees. An interview guide incorporating open-ended questions was pilot-tested on one surgeon and pathologist. A single investigator conducted all interviews by phone. Transcripts were analyzed independently by two investigators using a grounded approach,ho then compared findings to resolve differences.

**Results:**

Improvements in LN staging practice may have occurred largely due to educational presentations that created awareness, and self-initiated changes undertaken by pathologists. Executives that received performance data may not have shared this with staff, and opinion leaders engaged to promote compliance may not have fulfilled their roles. Barriers to change that are potentially amenable to quality improvement included perceptions about the practice (perceived lack of evidence for the need to examine at least 12 LNs) and associated responsibilities (blaming other profession), technical issues (need for pathology assistants, better clearing solutions and laboratory facilities), and a lack of organizational support for multidisciplinary interaction (little communication between surgeons and pathologists) or quality improvement (no change leaders or capacity for monitoring).

**Conclusion:**

Use of an exploratory approach provided an in-depth view of the way that numerous factors amenable to quality improvement influenced the adoption of new CRC LN staging recommendations. Continued interventions targeting physicians and executives, in the absence of a receptive organizational infrastructure, may be fruitless. Individualized rather than regional or punitive performance data, coupled with increased organizational capacity for change may stimulate greater surgical and organizational response to quality improvement. Descriptive or experimental studies are needed to test these hypotheses.

## Background

Treatment of colorectal cancer (CRC) involves surgical removal of the tumor and mesenteric lymph nodes (LN). Subsequent decisions about the need for additional treatment are partially based on whether pathologic findings demonstrate the presence of cancer in the LNs, since adjuvant chemotherapy confers a 15% absolute survival benefit for those with LN metastases [1]. Inadequate LN harvest has been associated with decreased survival, so several international cancer control agencies, including the National Cancer Institute, American Joint Committee on Cancer, and the International Union Against Cancer now recommend examination of a minimum of 12 LNs to optimize CRC staging [2,3].

We conducted a population-based study revealing that 27% of eligible CRC patients in the province of Ontario, Canada had an adequate LN assessment [4]. Dual efforts were consequently undertaken to improve this practice. In 2004 our group launched a randomized study (RCT) to examine the effect of engaging local surgeon or pathologist opinion leaders at participating hospitals on LN staging practice compared with a visiting educational presentation delivered to surgeons and pathologists (ISRCTN56824239) [5]. At the same time the provincial cancer agency (PCA) developed a series of CRC performance measures [6]. In 2005 the PCA distributed regional performance data to hospital CEOs and Chiefs of Surgery, including compliance with CRC LN staging recommendations. One year later the PCA reported that 58% of eligible CRC surgeries had 12 or more lymph nodes reported, a large change from the 27% revealed by population-based study, but this varied considerably across regions (range 33% to 92%) so there remained a need for ongoing quality improvement. Then the RCT was concluded and found that LN staging practice had improved in both the control and experimental arms of the trial [7]. There was no significant difference between the two arms in proportion of patients with 12 or more LN removed (64% control, 76% experimental). There was no consistent effect of academic status or hospital volume on LN retrieval rates. This study did not reveal how changes were achieved, or the factors influencing LN staging practice, information that could guide quality improvement efforts to further improve compliance.

The interventions employed by these initiatives, performance data, educational presentation and opinion leaders, represent distinct strategies for influencing care delivery. Public reporting of performance data has improved outcomes in some circumstances, for example, decreased mortality after cardiac surgery, but research suggests that such data has little effect on physician practice [8-11]. Managers are thought to be a better target for performance data because they wish to enhance their hospital's public image, particular if performance accountability agreements are in place [12]. Even so, it appears that managers may use performance data only when working in an institution that fosters a culture of quality, which suggests that organizational factors may be just as important as the information delivery mechanism [13].

Numerous interventions directed at physicians can improve compliance with practice recommendations but not across all conditions or settings of care, again highlighting the importance of organizational context [14]. For example, outreach visits involving educational presentations have been most useful for promoting prescribing practice [15]. Although opinion leaders appear to reduce non-compliance with desired practice, their effect appears comparable to that of educational visiting presentations or distribution of performance data, and studies have inconsistently described the actions of opinion leaders so the way they exert an effect in different contexts is not known [16]. Furthermore, the impact of these interventions has been largely examined under rigorous experimental conditions, which measure specific outcomes, but provide little insight on the circumstances underlying those findings, or guidance on how to best implement those strategies in the real world.

Several factors are known to enable or hamper the adoption of a new practice including the mechanism by which information about the practice is delivered, attributes of the practice, physician characteristics, and elements of the practice environment [17-19]. In particular, the importance of organizational capacity for facilitating change is increasingly evident [20-24]. A meta-analysis of data from 81 studies examining interventions designed to increase the use of screening services for colon, breast and cervical cancer by physicians found that organizational change strategies influenced use of services more than reminders, feedback of performance data, education, financial incentives, legislative action, or mass media campaign [25]. It is thought that organizational capacity includes the ability to identify, interpret, share and apply new knowledge or technology [26]. However, it is not known what organizational structures and processes optimize the adoption of different innovative practices.

Compliance with LN staging recommendations has improved, but inconsistently. The way in which different, theoretically-promising interventions, including performance data, educational presentation and opinion leaders may have had an effect on LN staging practice is unknown. It is possible that some organizations responded differently because they better processed and applied the information that was delivered through these interventions. No studies have investigated the factors that influence CRC LN staging practice. To reveal issues amenable to quality improvement we examined how interventions used to deliver LN staging information, and the characteristics of LN staging practice, physicians, and their environment contributed to changes in practice. The findings could be used to optimize the design of ongoing quality improvement efforts. They would also increase our understanding of how to better equip organizations to support changes in practice, and apply change interventions in a more effective manner.

## Methods

### Approach

With no previous research to understand perspectives on LN staging practice an exploratory approach was employed, involving qualitative analysis of interviews with relevant stakeholders, to gather detailed information about how individuals and their organizations responded to CRC LN staging information delivered through one of three interventions (performance data, educational presentation, opinion leader) [27].

### Setting

The study was conducted in community hospitals that had received CRC LN staging information through one of three interventions as identified with documentation from the PCA and RCT investigator. This included 11 educational presentation-only hospitals, nine opinion leader strategy hospitals, and ten performance data hospitals. Details about the interventions are described in Table 1. Ethical approval for the study was granted by the University of Toronto.

**Table 1:** Comparison of lymph node staging information delivery interventions

Aspect	Intervention		
	
	Randomized controlled trial		Performance data
		
	Educational presentation	Opinion leader strategy	
Responsible	Principal investigator	Principal investigator	Provincial cancer agency

Objective	Increase awareness of the need to assess a minimum of 12 LN for CRC staging through presentations delivered by a physician specializing in colorectal cancer surgery at participating hospitals	Increase awareness of the need to assess a minimum of 12 LN for CRC staging through presentations delivered by a physician specializing in colorectal cancer surgery, and engage an opinion leader at each hospital identified by peers to further promote this practice	Increase awareness of regional compliance with CRC LN staging recommendations in conjunction with performance-funding agreements to stimulate improvement
Timing	January to July 2004	January to July 2004	September 2005
Details	Presentations varied in length from one to four hours and included a lecture on importance of adequate LN assessment featuring graph of median LN counts from 1997 to 2000 for each hospital, and an interactive question and answer	• All hospitals received a presentation • Principal investigator met with the locally identified opinion leader to discuss possible barriers and solutions for improving CRC LN staging • Provided a pathology template for CRC, guidelines for what should be included in a pathology report for CRC, three copies of a poster with a picture of a colon with a number '12' watermarked over it, and three pocket cards on optimal pathology reporting • Six months following presentation the local opinion leader received a reminder package containing a peer-reviewed paper on LN clearing solutions, more pocket cards, and an invitation for further discussion if desired	• All hospitals received a presentation • Letter from PCA Head of Surgical Oncology noting regional compliance with CRC LN staging recommendations from June to August 2004.
Recipients	Surgeons and pathologists at 21 randomly allocated hospitals	Surgeons and pathologists at 21 randomly allocated hospitals with formally identified local opinion leader	CEOs and Chiefs of Surgery at 23 hospitals with PCA accountability agreements
Eligible sample for this study	Surgeons and pathologists at 11 hospitals that were exposed to presentation only and did not receive PCA performance data	Surgeons and pathologists at 9 hospitals that were exposed to presentation and opinion leader strategy but did not receive PCA performance data	Surgeons and pathologists at 10 hospitals were exposed to presentation only and received PCA performance data

### Sample Selection and Recruitment

Standard qualitative research principles were applied to determine recruitment methods and sample size [28]. First, all general surgeons and pathologists involved in CRC LN retrieval and assessment at eligible hospitals were identified from RCT investigator notes, searching a publicly available database of the physician licensing body, and asking study participants for the names of surgeons or pathologists at their hospital. They were invited to participate by regular mail. Consecutive, consenting physicians (convenience sampling) were recruited to represent both professions and different hospitals in a variety of health regions exposed to the three interventions (purposive sampling). The initial sampling target was one surgeon and one pathologist at each of two hospitals per intervention group for a minimum goal of 12 interviews. Hence, additional recruitment was restricted to hospitals with which initial responders were associated, and included faxing the study invitation to non-responders along with a follow-up phone call, and requesting referrals from those interviewed. For qualitative research the goal is to collect very detailed information from cases representative of different groups, rather than from a large overall number of cases. Analysis is concurrent with data collection, and sampling is deemed sufficient when no further new information emerges (informational redundancy) from successive interviews within groups (theoretical sampling) [28].

### Data Collection

Research on factors influencing changes in practice, including perceived attributes of the practice, physician characteristics, and organizational practice environment informed data collection [17-19] These are listed and defined in Table 2. Physician characteristics (specialization, sex, years in practice, and collegial networking) were collected by one-page survey included with the invitation mailing. Telephone interviews with surgeons and pathologists collected information on perceived attributes of LN staging practice (advantage, trialability, compatibility, uncertainty, complexity), practice environment (leadership, communication infrastructure, support for quality improvement), and how individuals or their organizations responded to the interventions to which they were exposed. An interview guide incorporating open-ended questions was developed and pilot-tested on one surgeon and pathologist, and refined based on their feedback. A single investigator (ARG), blinded to actual LN staging practice outcomes, conducted all interviews between February and June 2006, and an external professional transcribed audio-recordings.

**Table 2:** Theoretical framework of factors influencing adoption of a new practice

Concept	Component	Definition
LN staging attributes	Advantage	Obvious or proven benefit over previous practice
	Trialability	Control or autonomy over practice
	Compatibility	Easy to incorporate into practice; similar methods or philosophy
	Uncertainty	Facilitates individual clinical practice
	Complexity	Barriers to achieving recommended practice
Physician characteristics	Professional role	Surgeon, pathologist
	Sex	Male, Female
	Years in practice	Difference between date of interview and year of general surgery licensing
	Networking with colleagues	Frequency of participation in internal and external meetings for research, patient care, or planning
Organizational factors	Leadership	Active participation of executives and managers in quality improvement
	Communication infrastructure	Structures and processes that promote interaction and information sharing within and between departments
	Support for quality improvement	Resources for monitoring and implementing change

### Data Analysis

A grounded approach was used to analyze interview transcripts in an inductive, iterative manner [29]. Ideas were allowed to emerge from the data using the constant comparative technique, where transcripts are repeatedly read to identify and define important, unique themes relevant to study objectives [30]. These functions were managed without the assistance of analytic software, but each round of data analysis was thoroughly documented. To enhance reliability of the findings, two investigators (ARG, FCW) independently reviewed the transcripts and identified themes, then met to compare the findings and resolve differences through discussion. Transcript text was organized within tables by theme, intervention and type of physician to compare and interpret the factors influencing LN staging practice.

## Results

Interviews were conducted with 17 surgeons and nine pathologists whose characteristics are shown in Table 3. This included five surgeons and three pathologists from three of 11 eligible hospitals in three different health regions exposed to the educational presentation-only; six surgeons and three pathologists from five of nine eligible hospitals in three different health regions exposed to the opinion leader strategy; and six surgeons and three pathologists from three of ten eligible hospitals in three different health regions exposed to performance data. Hence, opinions were gathered from individuals at several different hospitals representing each of three information delivery interventions and seven different health regions. The way in which characteristics of LN staging practice, physicians and their organizations influenced response to educational presentation, opinion leader and performance data interventions is discussed here with representative quotes identified by an anonymous code.

**Table 3:** Participants interviewed by intervention, professional role and characteristics

Characteristic	Profession		Intervention		
	
	Surgeon	Pathologist	Educational presentation	Opinion leader strategy	Performance data
Whole group (n = 34)	17	9	8	9	9
Hospitals (n = 11)			3	5	3
Eligible	---	---	11	9	10
Participating	17	9	3	5	3
Profession					
Surgeon	17	---	5	6	6
Pathologist	---	9	3	3	3
Sex					
Male	14	5	6	7	6
Female	3	4	2	2	3
Age					
30–39	5	---	2	2	1
40–49	7	5	3	3	6
50–59	5	3	3	3	2
60–69	---	1	---	1	---
Year license					
Median (range)	1992 (1978,2004)	1988 (1967,1993)	1989 (1981,2004)	1993 (1967,2002)	1991 (1979,1999)
Collegial interaction					
Internal networking					
At least once per month	10	3	---	7	6
At least once per year	7	4	7	1	3
Never	---	2	1	1	---
External networking					
At least once per month	3	2	2	2	1
At least once per year	12	5	4	6	7
Never	2	2	2	1	1

### Awareness of the interventions

Awareness differed by professional and intervention group. Of 26 participating surgeons and pathologists, 25 (96%) were aware that 12 or more LN should be examined for accurate CRC staging. When asked how they learned about the LN staging recommendations surgeons and pathologists across all intervention groups (20/26, 77%) cited the educational presentation. Clinicians in the other intervention groups were less aware of the strategies or information that had been applied as part of those initiatives. Of nine surgeons and pathologists at hospitals exposed to the opinion leader strategy, four mentioned awareness of this initiative. Of nine surgeons and pathologists at hospitals receiving performance data, five were unaware of that information.

### Changes in practice

Profession, but not type of intervention exposure seemed to influence changes in LN staging practice. Most pathologists reported speaking with local colleagues – *I was the only pathologist who attended that meeting but disseminated that to other pathologists here *(P506), and those at other hospitals – *we spoke to neighboring hospitals and eventually found [a clearing agent] we were happiest with *(P553). Some hospitals hired additional personnel to accommodate new requirements for LN staging – *We hired a pathology assistant that grosses the majority of those specimens *(P562). Pathologists also reported changes to specimen preparation and analysis – *I'm slicing everything up in two millimeter sections throughout the entire specimen *(P573) and pathology reporting practices – *I put a comment down [in pathology report] that after careful assessment I can only find this many nodes *(P560) to ensure that surgeons were aware of the thoroughness of their assessment.

In contrast, few surgeons reported changes to their own surgical technique for extracting specimens that might be more likely to yield the minimum number of LNs – *the type of oncology operation I do is unchanged. I don't think that makes a big difference *(S537), or discussion with surgical colleagues about the need for quality improvement subsequent to interventions – *we haven't really discussed it amongst ourselves *(S505). Occasionally, if surprised by a low LN count, surgeons might ask the pathologist to *take another look *(537) or they might *review the specimen with the pathologist *(547).

### Factors influencing response

Perceptions about certain aspects of LN staging practice and associated responsibilities, technical issues related to surgery and pathology, and lack of organized communication and support for quality improvement may have been barriers to change. This did not appear to vary by profession or type of intervention.

#### Physician characteristics

Characteristics of surgeons and pathologists (Table 3) did not appear to influence response to CRC LN information, however, it is notable that half of the clinicians reported networking with internal colleagues only once per year or never, as this correlates with the reported limited interaction among and between the two professional groups engaged in LN staging.

#### Attributes of LN staging practice

Clinicians were asked several questions to elicit their views on the nature of LN staging practice, and understand how easily the new recommendations for retrieval and assessment of a minimum of 12 nodes could be adopted. There was some variability in responses by physician group but this primarily reflected professional role. Particularly relevant quotes are summarized in Additional File 1.

#### Advantage

All surgeons and pathologists understood the clinical implications of LN staging but many questioned the evidence behind the minimum 12 LN recommendation, and its association with patient outcomes.

#### Trialability

Participants said they had autonomy over this practice and could therefore modify it if necessary, but several blamed the lack of compliance and quality control on the other profession, or acknowledged that such beliefs existed.

#### Compatibility

Surgeons said they were already practicing appropriate surgical technique that would yield the required number of LNs. In contrast pathologists, who had reported several changes to their LN assessment and reporting practices, expressed concern over the associated increase in time and effort required to achieve recommended LN counts.

#### Uncertainty

Information on staging practice was not unwelcome. Pathologists said that receiving such information made them realize why the practice was clinically important. Surgeons said that LN counts provided them with feedback on the quality of their surgical technique. However, both groups expressed concern about potential punitive implications of non-compliance.

#### Complexity

Several technical and logistical aspects of LN staging practice could be influencing compliance with practice recommendations. Barriers of LN sample preparation and examination noted by pathologists included the need for additional help from pathology assistants, ineffectiveness of clearing solutions, and insufficient or unsafe laboratory facilities. Both surgeons and pathologists said that achieving a tissue sample containing a minimum of 12 LNs was dependent on surgical technique. However, compliance with staging recommendations may not be achievable in all patients given confounding factors such as physiology, comorbid conditions, cancer stage, and tumor location.

#### Practice environment

Participants were asked to describe formal or organized directives, means of communication, or quality improvement tools or initiatives that either directly or indirectly supported changes to LN staging practice. Little interaction occurred between surgeons and pathologists before, or after the interventions. Sometimes this was deliberate – *they don't tell me how to do my job, I'm not gonna tell them how to do their job *(502), and sometimes this was due to lack of structures to promote interaction – *in this hospital we don't have venues for interaction like rounds *(531), or incompatible scheduling – *all departments have rounds at the same time so it's not feasible to attend each *(566). As a result some surgeons said they *never speak with pathologists *(572) and were *not familiar with the process pathologists use *(S504) to examine LN specimens. Pathologists did not *know what constraints there are on the surgeon *(P573) when extracting the specimens. Neither group knew whether members of the other profession at their hospital had implemented any changes in their practice.

One hospital only had undertaken a formal quality improvement process by performing a prospective audit and sharing the data with both surgeons and pathologists. Otherwise, most surgeons and pathologists were unaware of whether they were meeting LN staging recommendations – *I don't have any idea how successful we are in achieving that goal *(S542). This information was not collected by their hospital and available to them electronically, nor did they have the time or means to conduct their own audits by reviewing individual patient medical records – *we don't have a mechanism in place to evaluate, out of all the cases I've done in the last year, how many met the standards *(P506).

When asked what strategies might improve LN staging practice, several surgeons and pathologists thought compliance would be enhanced with more manpower in the form of pathology assistants. Participants recommended greater interaction between surgeons and pathologists since LN staging involved a shared responsibility. They suggested that *tumor boards are probably a great help *(S502), or rounds or education programs *with surgeons and pathologists together *(S521). Both groups emphasized that comparative performance data was important, but recommended *more granular data, surgeon by surgeon, pathologist by pathologist *(S515) to identify more precisely where changes in practice were required, rather than the regional data that had been distributed by the PCA.

## Discussion

The purpose of this study was to examine how various factors influenced the outcome of different interventions implemented to increase compliance with LN staging recommendations, identify how this practice could be further improved, and generate knowledge on the structures and processes required in health delivery organizations to better adopt innovative clinical practices. No studies had qualitatively investigated this practice and, in taking an exploratory approach, several actionable issues were identified for practice and ongoing research.

Improvements in LN staging practice may have occurred largely due to educational presentations that created awareness about the new recommendations, and self-initiated changes undertaken by pathologists. There was little awareness of other interventions, so executives and managers that received performance data may not have shared this with staff, and opinion leaders engaged to promote compliance with the new practice may not have fulfilled their intended roles. Barriers to change that are potentially amenable to quality improvement included perceptions about the practice (lack of awareness of evidence for the need to examine a minimum of 12 LNs) and associated responsibilities (blaming other profession), technical issues (increased pathology effort, need for pathology assistants, better clearing solutions and laboratory facilities), and a lack of organizational support for multidisciplinary interaction (little formal or informal communication within and between surgical and pathology departments) or quality improvement (no leadership for change or capacity for monitoring).

Most surgeons in this study indicated they were not compelled to change their surgical technique but both surgeons and pathologists acknowledged that type of cancer and tumor location made LN retrieval challenging, and that the amount of tissue collected partially determined whether a minimum of 12 LN could be assessed. This study was not designed to examine individual lymph node recovery rates of participating surgeons, but future research might investigate whether and how surgical outcomes could be improved for cases with challenging tumor locations, and whether surgical decision-making or technical skill influence tissue retrieval [31]. Surgical judgment, largely based on personal assessments about technical ability, knowledge and experience, may not be amenable to modification using traditional continuing education due to a limited capacity of physicians to self-assess. Indeed, a number of studies have found sub-optimal performance in physicians who were the least skilled but the most confident in their technical ability [32]. A Cochrane review of 118 trials found that audit and feedback can be effective in improving clinical practice when individuals are able to compare their own performance with that of peers, data is provided periodically on an ongoing basis, and is delivered verbally or by senior colleagues [33]. Therefore, if it were found that surgical lymph node retrieval warranted improvement, individualized, comparative performance feedback could raise self-awareness of potential deficits in technical ability, and perhaps prompt alternative surgical approaches.

Participants said that more opportunities for fostering interaction between surgeons and pathologists must be created and supported. There is growing evidence that multidisciplinary collaboration improves health care team effectiveness and patient outcomes [34]. One option to consider is the tumor board or multidisciplinary cancer conference (MCC). These are regularly scheduled multidisciplinary meetings to prospectively review individual cancer patients and formulate appropriate management plans [35]. Several studies suggest that MCCs can improve compliance with practice recommendations but more research is required to understand how MCCs can be most effectively implemented [36-40].

Organizations monitoring, or attempting to influence performance cannot rely on distribution targets such as executives or clinical opinion leaders for communication of information to those engaged in the practice, or to implement change initiatives. Instead, performance reporting should be coupled with incentives for change. At this time the impact of pay-for-performance is unclear, and our participants were concerned about its implications [41]. They also requested individualized versus regional performance data. Research has shown that response to performance data is improved when the intent is explicitly non-punitive, and individual performance is compared to that of peers [42,43]. Additional research is necessary to understand alternative non-financial rewards. We learned that CRC staging based on 12 LNs is not possible in all patients due to physiologic characteristics, and nature and location of the cancer. Organizations monitoring LN staging must therefore ensure that performance data is adjusted for these factors, otherwise compliance could be under-reported.

However, even when coupled with appropriate incentives, interventions are likely to have little impact when the target lacks a receptive infrastructure. Institutional capacity for change may be a strong predictor for the adoption of innovations, and this includes the actions of managers [20-24]. For example, in American hospitals found to be high-performing for use of beta-blockers after acute myocardial infarction, managers ensured that resources were available to support changes, and actively promoted interdepartmental collaboration [44]. A survey of 1,784 American hospitals found that high involvement of managers in quality improvement significantly decreased inpatient mortality for several conditions [45]. Further investigations are required to describe and evaluate the structures and processes that contribute to quality improvement capacity.

Several limitations are inherent in the exploratory design of this study. First, the expressed opinions are particular to the involved participants, and their geographic and health system environment, and therefore may not be applicable to health professionals in other settings. However, such research is meant to generate hypotheses that can be tested through further descriptive research performed elsewhere. Second, recruitment in qualitative research typically involves a small number of participants that are not randomly selected, potentially biasing data collection. To mitigate this concern and optimize validity, we used purposive sampling so that a variety of possible opinions were collected from individuals representing several different hospitals within each intervention group and across different health regions. Furthermore, we achieved informational redundancy, both by profession and intervention group, underscoring the validity of our findings. Still, studies involving larger samples of hospitals and health professionals should be conducted to confirm the influence of opportunities for collegial interaction and organizational capacity for quality improvement on LN staging practice, as well as other clinical innovations.

## Conclusion

Use of an exploratory approach provided an in-depth view of the way that numerous factors amenable to quality improvement influenced the adoption of new CRC LN staging recommendations. In particular, lack of organizational infrastructure to coordinate and monitor changes in practice, and support multidisciplinary interaction may have limited the response to CRC LN staging recommendations. Individualized rather than regional or punitive performance data, coupled with increased organizational capacity for change may stimulate greater surgical and organizational response to quality improvement. Descriptive or experimental studies are needed to test these hypotheses.

## Competing interests

The author(s) declare that they have no competing interests.

## Authors' contributions

ARG obtained funding for the study, conceived and designed the study, acquired data, analyzed and interpreted data, and prepared the manuscript. FCW contributed to design of the study, assisted with data analysis and contributed to preparation of the manuscript. MAK contributed to design of the study, assisted with interpretation of the data, contributed to preparation of the manuscript. AJS assisted with interpretation of the data and contributed to preparation of the manuscript. All authors read and approved the final manuscript.

## Pre-publication history

The pre-publication history for this paper can be accessed here:



## Supplementary Material

Additional file 1 Table 4. Comparison of perceived lymph node staging attributes by intervention and profession has been uploaded as a separate file due to its length. The file is a Word document and has been named LNinterviews_BMCHSR_T4jan08.doc. It is referred to within the manuscript as Additional File 1. Click here for file
