# Supplementary material for: Multiple factors influence compliance with colorectal cancer staging recommendations: an exploratory study
Source: BMC Health Serv Res. 2008 Feb 6;8:34. doi: 10.1186/1472-6963-8-34 (PMC2270818; doi:10.1186/1472-6963-8-34)
Supplement: Additional file 1 — Table 4. Comparison of perceived lymph node staging attributes by intervention and profession has been uploaded as a separate file due to its length. The file is a Word document and has been named LNinterviews_BMCHSR_T4jan08.doc. It is referred to within the manuscript as Additional File 1. [file 1472-6963-8-34-S1.doc]

**Table 4 - Comparison of perceived lymph node staging attributes by intervention and profession**

| attribute | intervention | | | | | |
| --- | --- | --- | --- | --- | --- | --- |
|  | educational Presentation | |  | | educational Presentation | |
|  | surgeons |  | surgeons |  | surgeons |  |
| advantage |  |  |  |  |  |  |
| treatment planning | to give the most accurate staging (542)  chemotherapy improves survival and recurrence in patients who are stage III (538) | to determine if the patient should then go on to have chemotherapy (553) | if it’s node positive disease then that patient is getting chemotherapy (570) | to determine if the patient gets chemotherapy (545) | a stage III cancer would require chemotherapy (511)  adjuvant treatment makes a big difference in survival (505) | it's a certain stage then the patient will receive chemotherapy (562) |
| evidence lacking | no one has ever shown us survival benefit (502) | --- | the literature behind this is controversial (526) | whether you find 2 or 3 or 4 or 5, what difference does that make? I’ve never seen any data on that (573) | it’s probably a somewhat arbitrary number (516) | I don’t know how that came up; that number is double what I used to have to do (560) |
| trialability |  |  |  |  |  |  |
| freedom to implement | as a surgeon you can make an attempt to treat more tissue (542) | that’s up to us and our judgment (553) | I don’t think in our institution there are any restrictions (570) | I don’t think there are any constraints (573) | it's a technical issue which is purely in the hands of the individual doing the surgery (504) | --- |
| onus on other profession | you do the standard resection then its up to the pathologist (543)  surgeons have a tendency to say the pathologists aren’t looking at things well enough and the pathologists will say that the surgeons aren’t giving them enough material (502) | part of us succeeding in doing this depends on the surgeons (531) | I don’t think most of the variability’s by the surgeon (526)  if pathologists don’t achieve 12 it’s just because they’re lazy (521) | we have no control over the surgeon (528) | without me doing too much different they are reporting higher numbers (505)  there’s a tendency at surgical rounds to blame the pathologists, and a tendency at pathology rounds to blame the surgeons (515) | I am totally dependent on the specimen they give me (560) |

**Table 4 - Comparison of perceived lymph node staging attributes by intervention and profession, continued**

| attribute | intervention | | | | | |
| --- | --- | --- | --- | --- | --- | --- |
|  | educational Presentation | |  | | educational Presentation | |
|  | surgeons |  | surgeons |  | surgeons |  |
| compatibility |  |  |  |  |  |  |
| easily adopted or already in practice | the technique or type of oncology operation I do is unchanged (537) | --- | I don’t think it’s technically difficult (526) | --- | doing good anatomical dissection should give you the number of lymph nodes (505) | --- |
| adoption requires effort | --- | it’s much more time consuming (553)  for me it’s a question of the amount of time you spend on it (506) | --- | it can take up to an hour to search for lymph nodes sometimes (529) | --- | a limitation would be time (562) |
| uncertainty |  |  |  |  |  |  |
| meaningful performance indicator | lymph node status is how I gauge that I did the proper operation (538) | the more information we get, the more meaningful it is for us as opposed to just randomly searching (506) | use the lymph node as a surrogate for doing an adequate resection (526) | it’s a stimulus to have a serious look for them (573) | it demonstrates that an adequate surgical resection is being done (516) | I’m reassured that I’m doing a good job (560) |
| concerns about repercussions of non-compliance | it might have the potential for some problems (542) | I’d like to look at it as a recommendation as opposed to a standard (506) | is there a possibility for litigation (571)  it’s helpful if it’s meant as a guideline and not more than that (526) | patients may question the quality if they only have 10 nodes and know the recommendation to find 12 (545) | I don’t know if it has any medico-legal implications if you’re not able to retrieve 12 lymph nodes (507) | the surgeons felt that if they didn’t get the 12 lymph nodes that [the PCA] would remove colorectal surgery from this site (560) |

**Table 4 - Comparison of perceived lymph node staging attributes by intervention and profession, continued**

| attribute | intervention | | | | | |
| --- | --- | --- | --- | --- | --- | --- |
|  | educational Presentation | | opinion leader strategy | | performance data | |
|  | surgeons | pathologists | surgeons | pathologists | surgeons | pathologists |
| complexity |  |  |  |  |  |  |
| patient or tumor factors | it varies from person to person (542)  it depends on the location of the tumor (537) | often on the left-handed surgery no matter what you do it’s just not there (553) | I’ve had patients who are very small with very little mesentery (547)  it depends on the tumor location; sometimes you can’t see or feel them (572) | it’s a trade-off between doing damage to the bowel and collecting enough lymph nodes (573) | In a patient who is very elderly or very sick you just want to relieve the obstruction ore remove the tumor and so something as least invasive as possible (516)  some of the left colon cases can be a little more difficult (505) | different areas of the colon have different degrees of difficulty (566) |
| technical pathology factors | --- | clearing solutions have not worked and I’ve tried various recipes (506)  we have to do the gross ourselves, we don’t have assistants (531) | --- | the other pathologist doesn’t find solutions to be helpful (545)  we tried the clearing solution but it’s smelly and messy and delays things (573) | --- | we don’t have fumehoods and you’re supposed to have good ventilation (560)  we don’t have pathology assistants for lymph nodes and colons (553) |
| technical surgical factors | if I thought the problem was myself then I would make some technical adjustments (538) | if a surgeon makes a point of getting more sample and fat you’re able to get more lymph nodes (531) | if we’re doing a proper resection 12 lymph nodes is a piece of cake (521) | when they remove more mesentery we can find more lymph nodes (529) | it’s important that you’ve done a wide enough resection of tissue (504) | you’re not gonna get 12 nodes from a piece of bowel that’s no bigger than a toonie (566) |
